# METTL3-mediated HPV vaccine enhances the effect of anti PD-1 immunotherapy to alleviate the development of cutaneous squamous cell carcinoma^⋆^

**DOI:** 10.1016/j.abd.2023.05.006

**Published:** 2023-11-28

**Authors:** Yingjie Zhang, Yiru Wang, Shuping Guo, Hongzhou Cui

**Affiliations:** a Department of Dermatology, First Hospital of Shanxi Medical University, Taiyuan, China; b The First Clinical Medical College, Shanxi Medical University, Taiyuan, China; c Department of Dermatology, Taiyuan Maternity and Child Health Care Hospital, Taiyuan, China

**Keywords:** Carcinoma, squamous cell, CD8-positive T-lymphocytes, Papillomavirus vaccines

## Abstract

**Background:**

Cutaneous squamous cell carcinoma (cSCC) develops from epithelial keratinocytes by dysregulation of self-renewal and differentiation. Recent studies have found that the size and number of cSCC tumors gradually decrease or even disappear after HPV vaccination. However, the role of the HPV vaccine in the cSCC mechanism is poorly understood.

**Objective:**

The aim of this study is to investigate the effect and mechanism of the HPV vaccine in cSCC.

**Methods:**

Immunofluorescence was used to study the immune infiltrating cells in the tumor tissues of patients with cSCC. The effects of the HPV vaccine on cSCC cells and tissues were studied by Cell Culture, Real-time PCR, Western Blot, Cytotoxicity Assay, Enzyme-linked Immunosorbent Assay, m6A Blotting, CCK-8 Assay, m6A Ribonucleic acid Methylation Quantification and tumor transplantation.

**Results:**

The HPV vaccine enhanced the toxic effect of CD8^+^T cells on cSCC cells and promoted the secretion of multiple cytokines by CD8^+^T cells. In addition, HPV vaccines can increase tumor sensitivity to anti-PD-1 therapy by downregulating METTL3 in tumor tissue, with the combination of HPV vaccine and PD-1 monoclonal antibodies producing enhanced immune cell infiltration compared to PD-1 blockade alone.

**Study limitations:**

It is important to note the limitations of this study, including the small sample size, the construction of the mouse model, and the choice of HPV vaccine and PD-1 monoclonal antibody, which may limit the generalization of our findings to a wider population.

**Conclusions:**

It is hoped that this research will contribute to a deeper understanding of the role of the HPV vaccine in the treatment of cSCC. HPV vaccine is expected to become an important approach to alleviate the development of cSCC.

## Introduction

Cutaneous squamous cell carcinoma (cSCC) is a malignant tumor of the keratinocytes in the epithelial tissue, ranked second only to basal cell carcinoma in the incidence of non-melanoma skin cancers.^1^, ^2^ The pathogenesis of cSCC is still agnogenic, but there is increasing evidence that human papillomavirus (HPV) is involved in the pathogenesis of some squamous cell carcinomas, especially HPV16 or HPV18.^3^, ^4^ Low-risk HPV infections account for about 5% of all cancers worldwide, and high-risk HPV infections last longer than low-risk HPV infections.^5^ According to a meta-analysis, the risk of cSCC in immunocompetent individuals increased by 42% due to HPV infection.^6^

At present, the main treatment methods for cSCC are surgery and chemotherapy.^7^ When surgery is risky or impossible, it is still a challenge to find a safe and effective treatment. In 2017‒2018, three cases published in JAMA Dermatology showed a gradual decrease in the size and number of cSCC tumors or even their disappearance after HPV vaccination,^8^, ^9^ suggesting that the HPV vaccine plays an inhibitory role in the development of cSCC. Although the preventive utility of the HPV vaccine is well understood, its mechanism of action in the treatment of cutaneous malignancies is unclear.

In recent years, tumor immunotherapy has been expected to become a new approach in the field of tumor therapy.^10^ CD8^+^T cells are the most important regulatory cells in tumor-acquired immunity, which mediates anti-tumor immunity by directly killing cancer cells, so activation of CD8^+^T cells may be the key to immunotherapy for cSCC. Studies have shown that Programmed Death ligand-1 (PD-1) is highly expressed in CD8^+^T cells in cSCC and blocking PD-1 with monoclonal antibody is one of the effective methods.^11^, ^12^ PD-1 inhibitors can block the binding of PD-1 and PD-L1, resulting in the transduction of immunosuppressive signals in the PD-1/PD-L1 pathway, and then restore and promote the immune killing function of CD8^+^T cells,^10^, ^13^ making them play a powerful anti-tumor role. It has been reported that the HPV16 L2E7E6 fusion protein vaccine called Tissue Antigen ‒ Cervical Intraepithelial Neoplasia and PD-1 blocker have synergistic effects in TC-1 tumors to better control tumors.^5^, ^14^, ^15^

Epigenetics has evolved into a hot topic in scientific research.^2^ N6-methyladenosine (m^6^A) is a reversible methylation modification at the sixth N atom of adenine,^16^, ^17^ which has been demonstrated to play an important role in many biological processes, such as adipogenesis, sperm germinal, tumor, and other diseases.^18^, ^19^, ^20^ However, to date, there have been few studies on how m^6^A modifications affect the progression of cSCC. Although m^6^A has been the focus of many studies in recent years, a comprehensive understanding of m^6^A has yet to be realized, and the underlying mechanisms of m^6^A modification in cancer should be further investigated. Recent studies have shown that m^6^A modification is closely related to tumor immune microenvironment remodeling, which may influence tumor development.^21^

In this study, the authors hypothesized that the use of HPV vaccination with anti-PD-1 injection could significantly increase the number of tumor-infiltrating CD8^+^T cells in mice with cSCC, eliciting a significant immune response that would synergize with PD-1 blockade and would provide insights into the potential impact of HPV vaccination on currently understudied HPV-associated malignancies. The idea that the HPV vaccine enhances the anti-PD-1 effect via METTL3 for the treatment of cSCC was also proposed through anthropological specimens, cytological experiments, and animal experiments. The present findings reveal a previously unrecognized mechanism of mRNA methylation in cSCC sensitized to PD-1 blockade, thus providing a potential therapeutic avenue for this malignant disease.

## Materials and methods

### Tissue samples

For the collection of cSCC samples from patients, analyses were performed with the approval of the Ethical Committee of the First Hospital of Shanxi Medical University. cSCC tissue was collected from 10 patients after surgery and the surgical details of those 10 patients are shown in Table 1. The pathologist confirmed the type of pathology in the sample.

**Table 1:** Details of human studies.

Number	Age	HPV vaccination	Site	Diameter(cm)	Procedure
1	56	N	Anogenital areas	2.3	Mohs
2	34	N	Head	1.4	Standard excision
3	45	Y	Head	1.6	Standard excision
4	36	N	Head	0.8	Standard excision
5	65	Y	Neck	1.4	Standard excision
6	46	N	Head	1.5	Standard excision
7	48	Y	Vermillion lips	1.8	Standard excision
8	39	N	Head	3.2	Mohs
9	47	Y	Pretibial areas	1.7	Standard excision
10	51	Y	Head	1.2	Standard excision

### Immunofluorescent staining

Tumor sections embedded in paraffin were dried at 90 °C for 4 h, dewaxed in xylene, and then rehydrated in graded ethanol solutions. Cooled tissue sections were immersed in 0.3% hydrogen peroxide solution for 15 min to block endogenous peroxidase activity, rinsed with PBS for 5 min, and blocked with 3% BSA solution at room temperature for 30 min. After washing by PBS, the sections were incubated with antibodies (Abcam, Cambridge, MA, USA) for 1 hour in 1% BSA blocking buffer, followed by incubated for 1.5 hours of secondary antibody conjugated with Alexa Fluro 549 (Thermal Fischer) and DAPI. Fluorescence images were obtained using a Zeiss LSM510 confocal microscope, equipped with a 63× immersion objective.

### Cell culture

A431(Human cSCC cell) and SCC7 (mouse SCC cell) were obtained from the Cell Bank of Shanghai Institute of Cell Biology (Shanghai, China). These cells were maintained in a DMEM (Thermo Fisher Scientific, Waltham, MA, USA), which is supplemented with 10% FBS and 1% penicillin-streptomycin. Cells were cultured in a humidified incubator at 37 °C with 5% CO_2_ (Sanyo Osaka, Japan).

### Preparation of CD8^+^T cells

Peripheral blood mononuclear cells from blood donors were isolated by using the Ficoll gradient method, and CD8^+^T cells were isolated from 1.25 × 10^7^ PBMCs by using the Easy Sep Human CD8^+^T Cell Isolation Kit.

### Real-time PCR

Extraction of total RNA was performed using TRIzol reagent (Invitrogen, Carlsbad, CA, USA) and reverse-transcribed using the cDNA Synthesis kit (Takara, Japan). The quantity and density of the RNA were verified by a spectrophotometer. QRT-PCR was performed using the SYBR Green Master MIX Kit (Takara, Japan) according to the manufacturer’s instructions. The assays were performed in triplicate and relative gene expression was determined by using the 2^-ΔΔCt^ method.

METTL3 forward: 5’- CATCCGTCTTGCCATCTCTAC-3’

METTL3 reverse: 5’- GACCTCGCTTTACCTCAATCA-3’

METTL14 forward: 5’- GCATCACTGCGAATGAGAAATG-3’

METTL14 reverse: 5’- CAGAACCGCACCAGAGAAATA-3’

RMB15 forward: 5’- GCCCAACTCAAGATCACTCA-3’

RBM15 reverse: 5’- AGCCAGGAGAATGGCATAAC-3’

WTAP forward: 5’- AATGGTAGACCCAGCAATCAA-3’

WTAP reverse: 5’- CGTAAACTTCCAGGCACTCA-3’

VIRMA forward: 5’- GAGGCGGATCCTTTGAGTTT-3’

VIRMA reverse: 5’- AACGACCTGAGAGAGGGATAG-3’

FTO forward: 5’- AGAGCGGGAAGCTAAGAAAC-3’

FTO reverse: 5’- GCCACTGCTGATAGAACTCAT-3’

ALKBH5 forward: 5’- GTGGGTATGCTGCTGATGAA-3’

ALKBH5 reverse: 5’- TCGCGGTGCATCTAATCTTG-3’

### Western blot

To extract total proteins, cell lysates were obtained using RIPA buffer (Beyotime, Shanghai, China) supplemented with phosphatase and protease inhibitors (Yeasen, Shanghai, China). A total of 15 μL protein was injected into a Bis-Tris SDS/PAGE gel and transferred to PVDF membranes. After blocking with 5% BSA, the membranes were incubated with primary antibody overnight at 4 °C. Membranes were then exposed to the secondary antibody for 60 min. The strips were incubated with an ECL kit and analyzed with an imaging system. Densitometric analysis was performed by using Adobe Photoshop CS6.

### Cytolytic assay

Purified CD8^+^T cells were obtained by negative selection. SCC was added to purified CD8^+^T cells at a 1:10-50 ratio and cultured in 48-well trays (Costar 3548, Cambridge, MA) in RPMI 1640. IL-2 (50 units/mL, Chiron, Emeryville, CA) was added to each well beginning on day 1 and every 2 to 3 days thereafter. SCC cells were harvested on day 7 and assayed for lytic activity in triplicate using CCK-8 assay.

### Enzyme-linked immunosorbent assay

Enzyme-linked immunosorbent assay (ELISA) to detect cytokines was performed with kits (Boster Bio, Wuhan, China).

### CCK-8 assay

A total of 10^3^ cells were inoculated into each well of 96-well plates. At each time point (24 h, 48 h, 72 h, and 96 h), 10 μL CCK-8 solution (Yeasen, Shanghai, China) was added to the sextuplicate wells. The wells were incubated for 3 h, and the absorbance of each well was determined at 450 nm.

### In vivo assays

All of the animal experiments were approved by the Animal Experimentation Ethics Committee of the first Hospital, Shanxi Medical University. Eight-week-old C57BL/6 mice were maintained according to the guidelines of the 3Rs (Replacement, Reduction, and Refinement). A total of 1 × 10^7^ SCC7 cells resuspended in 100 μL of PBS were inoculated subcutaneously in the left flank of the mice. When the tumor volume reaches 50‒100 mm^3^, anti-PD-1 antibody (200 μg/mouse) or combined HPV vaccine (0.25 μL/mouse) was injected. After the tumor was detected, tumor size was measured every 5 days by a vernier caliper and tumor volume was calculated as volume (cm^3^) = L × W^2^ × 0.5 with L and W representing the largest and smallest diameters, respectively.

### m^6^A RNA methylation quantification

The m^6^A RNA Methylation Assay Kit (Abcam, ab185912) was used to evaluate the content of m^6^A in total RNA.

### Dot blot

The poly(A) + RNAs (400 ng) were double-diluted and spotted onto a nylon membrane (GE Healthcare, USA). Briefly, the membranes were then UV crosslinked, blocked, incubated with m^6^A antibody and horseradish peroxidase-conjugated anti-rabbit IgG, and finally detected with a 3,3’-diaminobenzidine peroxidase substrate kit. The same 400 ng poly(A) + RNAs were spotted on the membrane, stained with 0.02% Methylene blue in 0.3 M sodium acetate for 2 h, and washed with ribonuclease-free water for 5 h.

### Statistical analysis

Statistical analyses were performed using the SPSS software version 19.0 (SPSS, Inc., Chicago, IL) or GraphPad Prism 7.0 (GraphPad Software, USA) as previously described. The values are presented as the mean ± Standard Deviation (SD). For comparisons, Student’s *t*-test and one-way ANOVA test by Least Significant Difference test were performed, as appropriate; p < 0.05 was considered statistically significant.

## Results

### HPV vaccination enhances the infiltration of immune cells in human cSCC tissues

**Figure 1:**
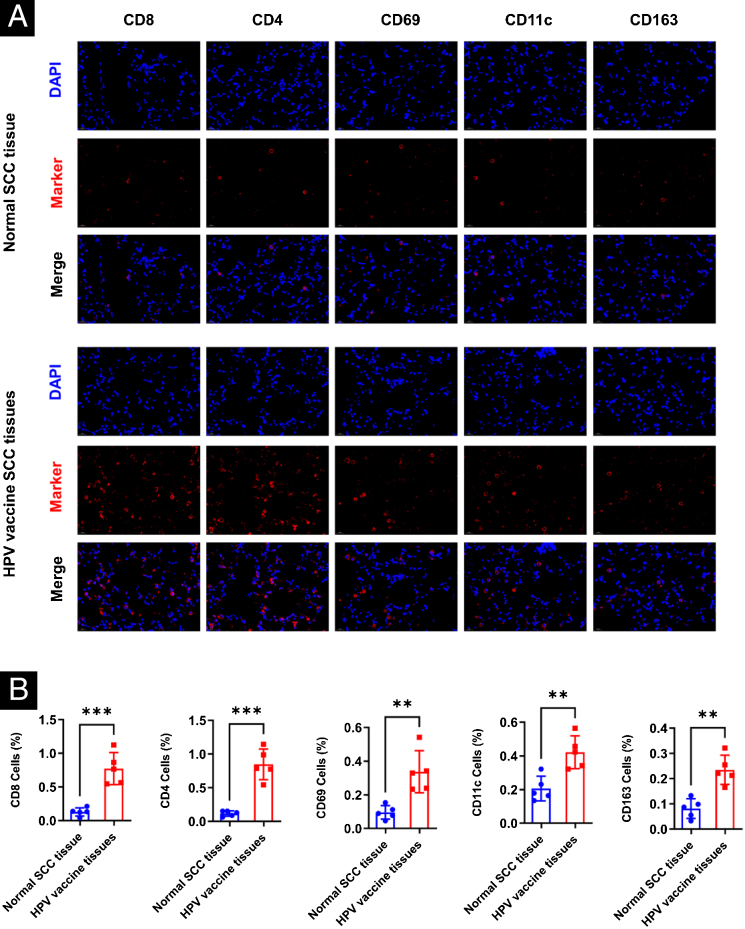
Differences in immune microenvironment in cSCC tissues of HPV vaccinated and unvaccinated patients. (A) Differences in immune microenvironment (CD8, CD4, NK, DC, Macrophage) in cSCC tissues; (B) Image J statistical analysis of the percentage of positive immune cells. **p < 0.01, ***p < 0.001.

The authors selected five cases each of HPV-vaccinated and non-HPV-vaccinated cSCC patients' tumor tissues in order to determine the effect of the HPV vaccine on immune cells in human cSCC tissues, and analyzed the infiltration of CD8, CD4, NK, DC, and Macrophages in cSCC tumors by immunofluorescence. There was a statistical significant difference between the two groups in the five types of immune cells. The expression of CD8, CD4, CD69, CD11c, and CD163 in the tumors of humans in the vaccinated group was significantly increased (Fig. 1A‒B).

### HPV vaccine enhances the killing ability of CD8^+^T cells

**Figure 2:**
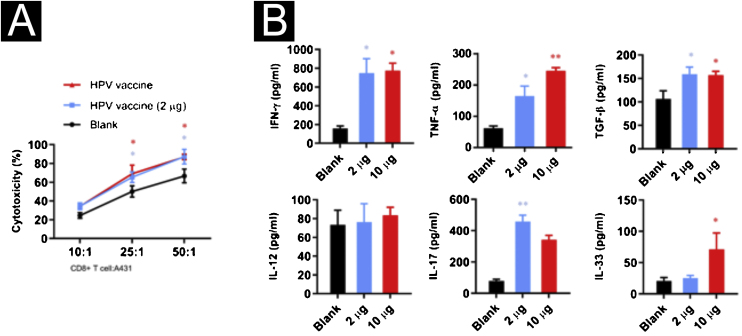
Effect of HPV vaccine on CD8^+^T cell activity in cSCC. (A) CD8^+^T cell killing assay on A431 cells; (B) secretion of inflammatory factors by HPV vaccine stimulation on CD8^+^T cells. *p < 0.05, **p < 0.01, ***p < 0.001.

To investigate the effect of the HPV vaccine on CD8^+^T cell activity, the authors co-cultured CD8^+^T cells with A431 cells at 10:1, 25:1 and 50:1 ratios and added 2 μg or 10 μg of bivalent HPV vaccine Cervarix (GSK, 0.5 μg/mL). CD8^+^T cells showed higher cytotoxic activity after adding the HPV vaccine, however, the differences in the killing activity of the HPV vaccine at different concentrations on A431 cells were not statistically significant at a certain potency-target ratio (Fig. 2A). To explore the effect of HPV vaccine on cytokine secretion in the co-culture system, the authors examined IFN-γ, TNF-α, TGF-β, IL-17, IL-12, and IL-33 in the supernatant, and the secretion of IFN-γ, TNF-α, TGF-β, and IL-17 increased significantly after the addition of 2 μg of HPV vaccine, while the secretion increased after the addition of 10 μg of HPV vaccine was IFN-γ, TNF-α, TGF-β, and IL-33 (Fig. 2B).

### HPV vaccine combined with anti-PD1 monoclonal antibody treatment inhibits tumor development in mice in vivo

**Figure 3:**
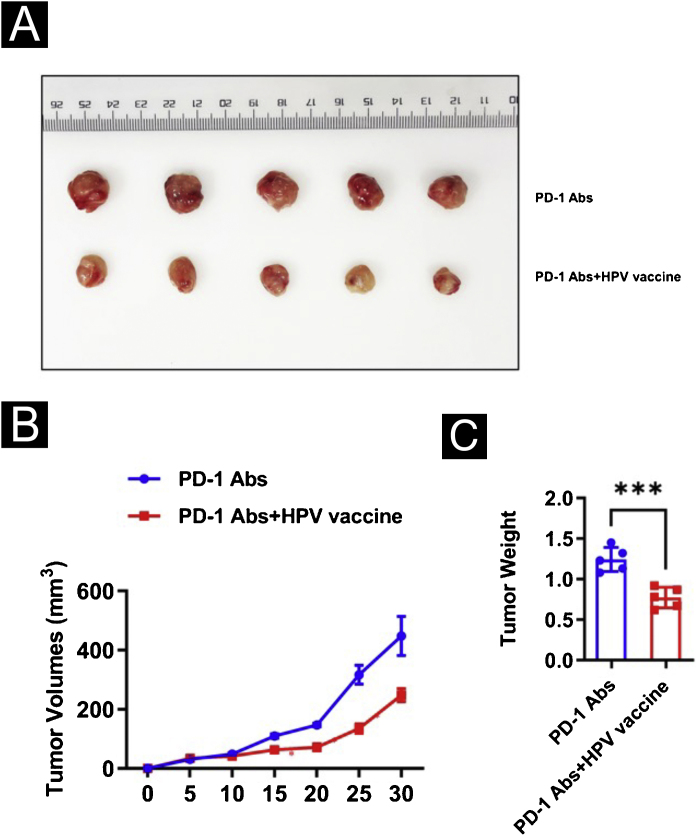
Impact of HPV vaccine combined with PD-1 monoclonal antibody for cSCC. (A) Tumor picture; (B) Volume change; (C) Weight change. ***p < 0.001.

**Figure 4:**
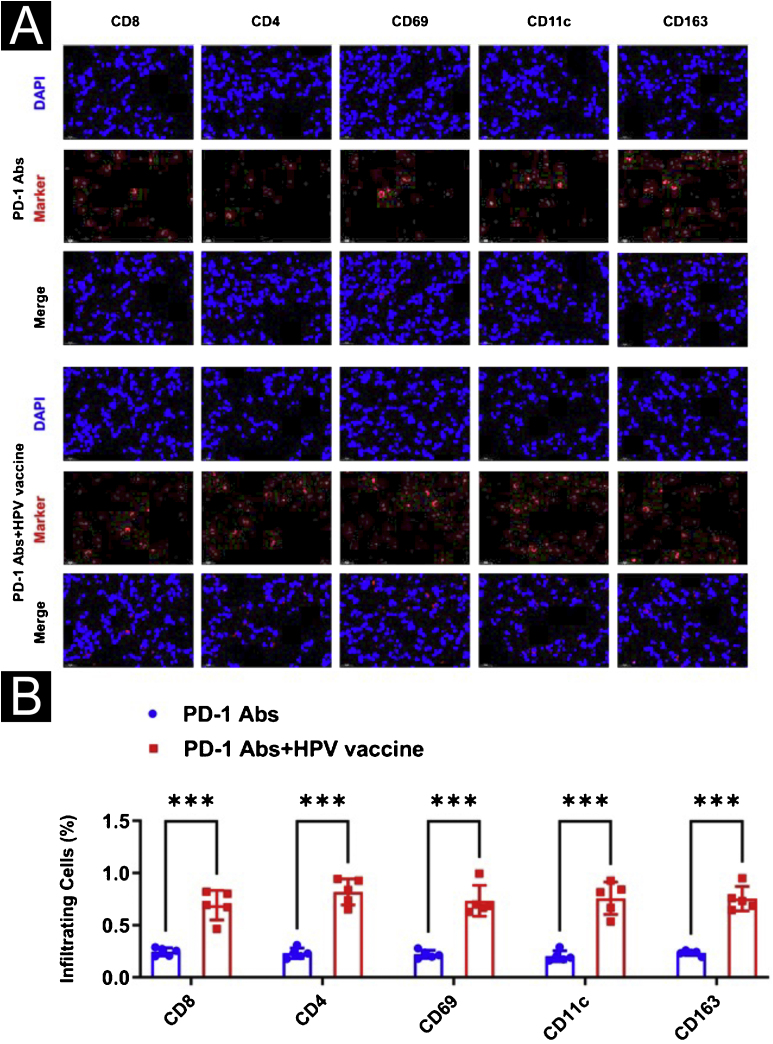
Effect of HPV vaccine combined with PD-1 monoclonal antibody on immunofluorescence in mice. (A) Immunofluorescence observation of immune microenvironment differences (CD8, CD4, NK, DC, Macrophage). (B) Image J statistical analysis of the percentage of positive immune cells. ***p < 0.001.

To further analyze the effect of the HPV vaccine on cSCC at the animal level, the authors hypothesized that the addition of the HPV vaccine would enhance the immunogenicity and antitumor effects of PD-1 treatment in mice. To test this hypothesis, the authors divided 10 mice with cSCC into two groups of 5 mice each and injected PD-1 Abs (10377-M94, SinoBiological) or PD-1Abs+HPV vaccine. The authors did not set up a no-treatment group reflecting the therapeutic effect of PD-1 on cSCC, because it has been concluded that PD-1 monoclonal antibody has a significant inhibitory effect on cSCC in other studies.^22^, ^23^, ^24^, ^25^ To monitor the antitumor response, tumor volume was measured every 5 days, and mice were executed at day 30, tumor tissue was isolated and weight was measured (Fig. 3A). Compared to PD-1 Abs, tumors in the PD-1Abs+HPV vaccine group were reduced in weight (Fig. 3B) and volume (Fig. 3C) at day 30, which demonstrated that stable regression was observed on tumors in all groups. Immunofluorescence analysis of mouse tumor tissues observed a significant increase in intratumoral infiltration of CD8, CD4, NK, DC, and Macrophage in PD-1 monoclonal antibody combined with HPV vaccine mice (Fig. 4A‒B).

**Figure 5:**
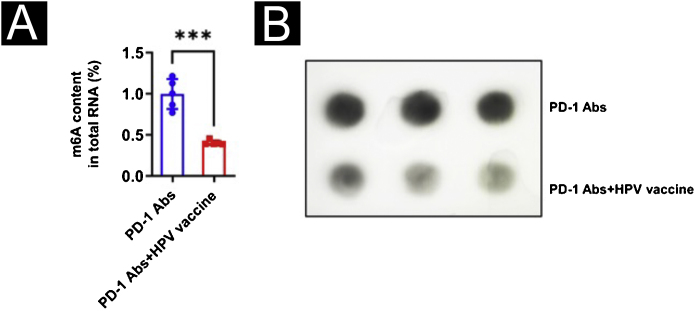
m^6^A mRNA and protein expression in tumor tissues. (A) Colorimetric analysis of m^6^A content in tumors; (B) Dot Blot to verify m^6^A content in tumors. ***p < 0.001.

**Figure 6:**
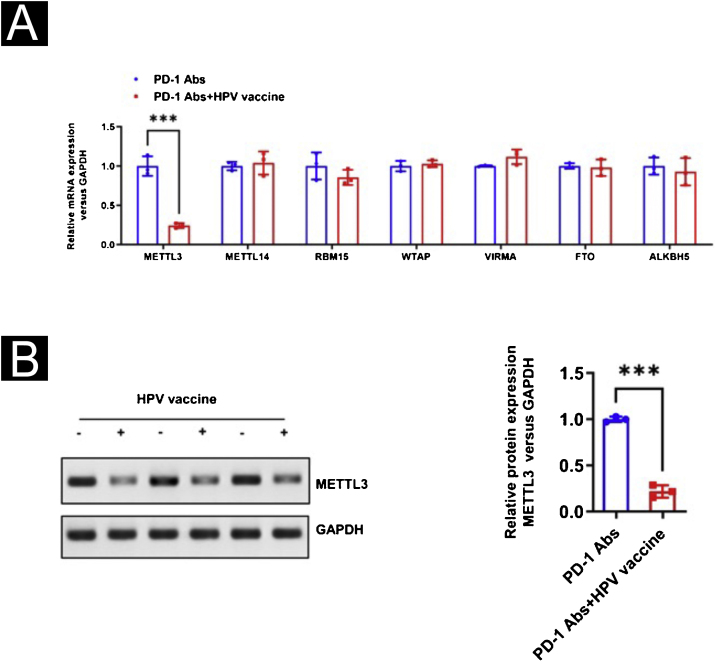
Selection of targets with aberrant m6A expression. (A) Real-time PCR to screen the expression of m^6^A-related methylesterase and demethylase in tumor tissues; (B) Western Blot to verify the difference of METTL3 expression. ***p < 0.001.

With the purpose of observing the effect of the HPV vaccine on m^6^A in tumor tissues, the authors analyzed the m^6^A content in mouse tumor tissues using a colorimetric method and Dot blot and found that the m^6^A content in the PD-1Abs+HPV vaccine group was significantly lower than that in the PD-1 Abs group (Fig. 5A‒B). The authors further screened the expression of m^6^A methylesterase and demethylase by qPCR, and the results were shown in 3H. METTL3 was significantly downregulated in the presence of HPV vaccine, while the differences of METTL14, RBM15, WTAP, VIRMA, FTO, and ALKBH5 were not statistically significant (Fig. 6A). The downregulation of METTL3 expression at the protein level in PD-1Abs+HPV vaccine group was further verified by Western blot (Fig. 6B).

### METTL3-mediated HPV vaccine enhances susceptibility to cSCC immunotherapy

**Figure 7:**
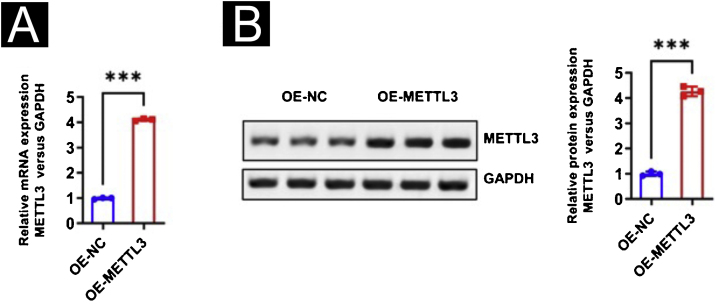
Identification of METTL3 overexpression. (A‒B) Real-time PCR and Western Blot verified the efficiency of METTL3 overexpression. ***p < 0.001.

**Figure 8:**
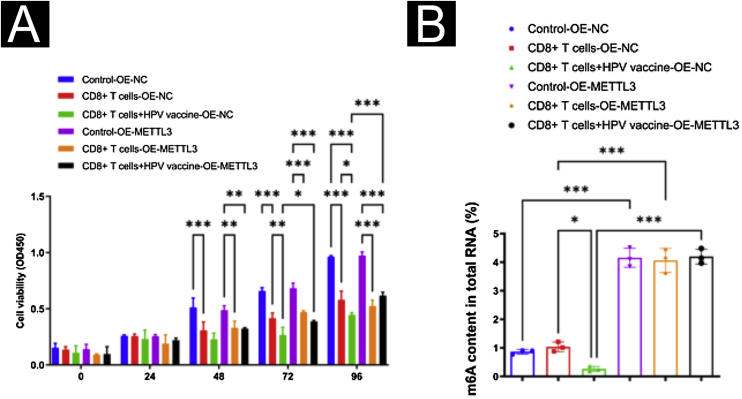
Mechanisms of HPV-enhanced susceptibility to SCC immunotherapy. (A) CCK8 assay revealed that HPV vaccine significantly promoted the suppressive effect of CD8^+^T cells on A431 cells, while METTL3 overexpression attenuated the suppressive effect of HPV vaccine treatment on A431 cells by CD8^+^T cells; (B) m^6^A assay revealed that HPV vaccine significantly reduced the total m^6^A content in the high CD8^+^T cells + A431 cells co-culture system, while METTL3 knockdown significantly abolished this effect. *p < 0.05, **p < 0.01, ***p < 0.001.

To further study the effect of the HPV vaccine on METTL3 expression in cSCC, the authors overexpressed METTL3, qPCR and Western blot were performed to detect the overexpression efficiency at mRNA and protein levels (Fig. 7A‒B). CCK-8 assay revealed that there was no significant difference in cell activity between the six groups within 24 hours of incubation. After 96 hours of culture, cell activity was significantly reduced when co-cultured with CD8^+^T cells and A431 cells compared with the control group, and further reduced in A431 cell activity after the addition of HPV vaccine, with statistical differences between all groups. After overexpression of METTL3, it still showed the inhibitory effect of CD8^+^T cells and HPV vaccine on A431 cell activity, and A431 cell activity was significantly higher in the CD8^+^T cells+HPV vaccine-OE-METTL3 group than in the CD8^+^T cells+HPV vaccine-OE-NC group (Fig. 8A). Also, the authors examined m^6^A in each group and found that HPV vaccine significantly reduced the total m^6^A content in the co-culture system, while METTL3 overexpression significantly abolished this effect. Interestingly, the m^6^A content in the CD8^+^T cells+HPV vaccine-OE-NC group was not statistically significantly different from Control -OE-NC, while the m^6^A content in the CD8^+^T cells -OE-NC group was elevated compared to Control -OE-NC, although there was no statistical difference (Fig. 8B).

### Overexpression of METTL3 abolishes HPV vaccine inhibition of cSCC in vivo

**Figure 9:**
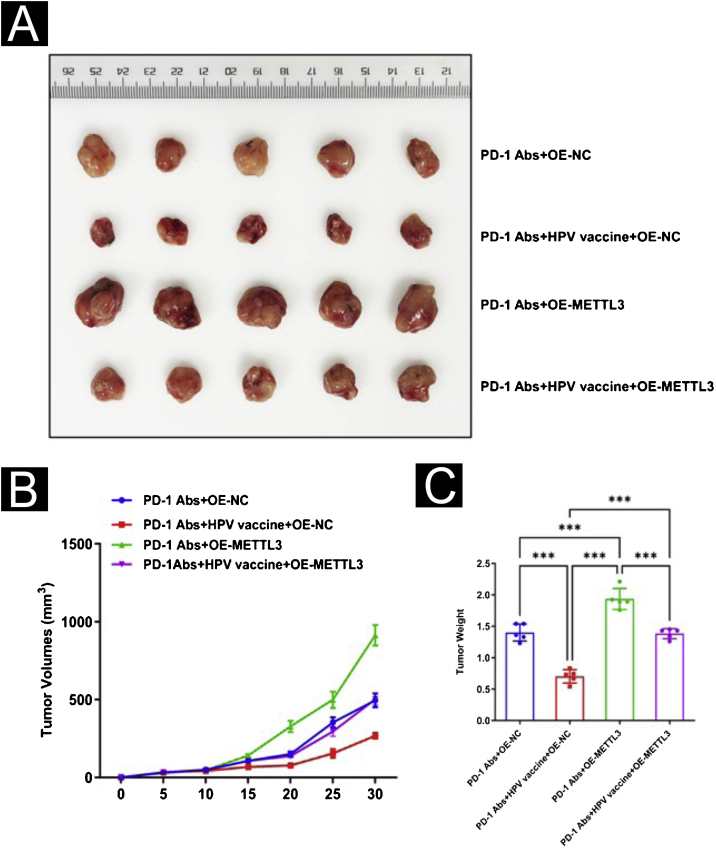
Animal-level validation of METTL3 overexpression abolishing HPV vaccine to enhance the sensitivity of cSCC mice treated with PD-1 monoclonal antibody. (A) Tumor picture; (B) volume change; (C) weight change. ***p < 0.001.

**Figure 10:**
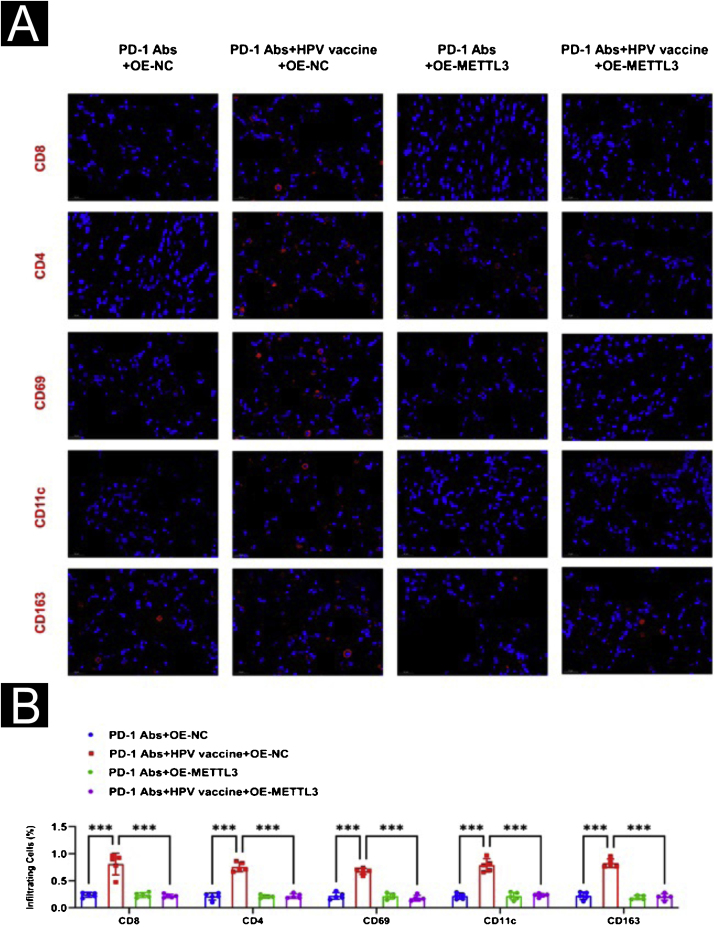
Effect of overexpression of METTL3 on combination therapy in immunofluorescence. (A) Immunofluorescence observation of immune microenvironment differences (CD8, CD4, NK, DC, Macrophage); (B) Image J statistical analysis of immune cell positive ratio. ***p < 0.001.

For further evaluation of METTL3 in the HPV vaccine against csCC, xenograft tumor experiments were performed. The authors divided the mouse models of cSCC into 4 groups of 5 mice each and gave PD-1 Abs+OE-NC, PD-1 Abs+HPV vaccine+OE-NC, PD-1 Abs+OE-METTL3, and PD-1 Abs+HPV vaccine+OE-METTL3 treatments respectively. In the combination of anti-PD-1 antibody and HPV vaccine, tumor volume and weight were significantly reduced, and co-treatment with HPV vaccination and anti-mouse PD-1 antibody resulted in complete inhibition of tumor growth, while the inhibitory effect of anti-PD-1 antibody on tumor volume and weight was attenuated after overexpression of mettl3 (Fig. 9A‒C). Histologically, it was found that the PD-1 Abs+HPV vaccine+OE-NC group had significantly more intratumoral infiltrates of CD8, CD4, NK, DC, and Macrophages than the other groups (Fig. 10A‒B).

## Discussion

Immunosuppression is mainly manifested by diminished or absent proliferative capacity of T lymphocytes and dysfunctional cytokine secretion.^26^, ^27^ CD8^+^T cells are immune cells that can recognize and kill cancer cells, considered to have tumor-eradication potential.^28^ The present study found that the HPV vaccine enhanced the toxic effects of CD8^+^T cells on cSCC. CD8^+^T cells are multiple cytokine producers and the HPV vaccine resulted in increased secretion of IFN-γ, TNF-α, TGF-β, IL-17, IL-33 by CD8^+^T cells. Yunyan Sun concluded that both cervicovaginal vaccinations with TA-HPV could induce a strong local HPV-16E7 antigen-specific CD8^+^T cell immune response.^29^ The present results suggest that after HPV vaccination in humans, the body produces a certain amount of specific T-cell immune response along with antigen-antibody response, and the resulting cytokines play a role in suppressing cutaneous squamous carcinoma cells, suggesting that HPV vaccine may be better at stimulating antitumor immune response.

RNA modifying enzymes are crucial for tumor survival in the process of immunotherapy, and m^6^A modifications play oncogenic roles in many cancers.^30^ The present results showed that overexpression of METTL3 in cSCC cells increased the level of m^6^A modification in cSCC cells, which confirmed the study of Renpeng Zhou from the side,^31^ indicating that METTL3 maintained m^6^A modification in tumor cells and promoted the proliferative activity of cancer cells in cSCC. Therefore, the authors conclude that the HPV vaccine inhibits the development of squamous carcinoma by down-regulating METTL3, probably because the downregulation of METTL3 expression increases the sensitivity of tumor cells to cytokines. Lingling Wang suggested that high METTL3 expression status was negatively correlated with tumor immune cell infiltration,^32^ likewise, the results showed that the immune infiltrating cells in cSCC tissues were significantly reduced after METTL3 overexpression in the animal experiment of this study.

METTL3 may be a potential target for clinical treatment of cSCC.^33^ Eliza Yankova demonstrated that a METTL3 inhibitor (STM2457) inhibits the carcinogenic effects caused by METTL3 enzyme overexpression in both in vitro cellular and in vivo animal experiments.^34^ lingling Wang demonstrated that depletion of the methyltransferases Mettl3 and Mettl14 enhanced response to anti-PD-1 treatment in colorectal cancer and melanoma.^32^ Overexpression of METTL3 followed by treatment with PD-1 monoclonal antibodies did not show a significant trend of tumor regression in the present results, whereas the HPV vaccine has the potential to act as a METTL3 inhibitor, and the combination with PD-1 monoclonal antibodies for cSCC played a synergistic role to further slowdown the tumor growth.

There is an urgent need to discover precise and effective targets for current therapeutic approaches in cSCC. The main finding of this study was that HPV vaccine treatment of squamous carcinoma is correlated with METTL3 expression and also inhibited the formation of an immunosuppressive tumor microenvironment. There are certain shortcomings in this study, the sample size of clinical samples is small, and sufficient samples may need to be collected for future studies, while further use of m^6^A sequencing and transcriptome sequencing may be needed to deeply explore the molecular regulatory mechanisms of METTL3-mediated HPV vaccine treatment for cSCC. In conclusion, the present experiments suggest that METTL3 is a promising target for the treatment of cSCC that can be downregulated by HPV vaccines.

## Conclusions

The HPV vaccine enhances the sensitivity of anti-PD-1 treatment by down-regulating METTL3 in cutaneous squamous cell carcinoma. The combination of HPV vaccine and PD-1 monoclonal antibody treatment produces an immune infiltration-enhancing effect, resulting in better tumor control and promoting tumor clearance. Overall, this study provides some reference value for the in-depth exploration and research of the HPV vaccine. In future studies, research on the mechanistic aspects of METTL3 should be further strengthened.

## Financial support

None declared.

## Authors’ contributions

Yingjie Zhang: The study concept and design; data collection, or analysis and interpretation of data; writing of the manuscript; data collection, analysis and interpretation.

Yiru Wang: Data collection, or analysis and interpretation of data; data collection, analysis, and interpretation.

Shuping Guo: Statistical analysis; data collection, analysis, and interpretation; effective participation in the research guidance.

Hongzhou Cui: Critical review of important intellectual content; effective participation in the research guidance; critical review of the literature; final approval of the final version of the manuscript.

## Conflicts of interest

None declared.
